# Identification and Characterization of Major Bile Acid 7α-Dehydroxylating Bacteria in the Human Gut

**DOI:** 10.1128/msystems.00455-22

**Published:** 2022-06-23

**Authors:** Kyung Hyun Kim, Dongbin Park, Baolei Jia, Ju Hye Baek, Yoonsoo Hahn, Che Ok Jeon

**Affiliations:** a Department of Life Science, Chung-Ang University, Seoul, Republic of Korea; University of California, San Diego

**Keywords:** 7α-dehydratase, BaiE, IBD, bile acids, human gut microbiota, metabolic pathways, sequence similarity networks

## Abstract

The metabolism of bile acids (BAs) by gut bacteria plays an important role in human health. This study identified and characterized 7α-dehydroxylating bacteria, which are majorly responsible for converting primary BAs to secondary BAs, in the human gut and investigated their association with human disease. Six 7α-dehydratase (BaiE) clusters were identified from human gut metagenomes through sequence similarity network and genome neighborhood network analyses. Abundance analyses of gut metagenomes and metatranscriptomes identified a cluster of bacteria (cluster 1) harboring *baiE* genes that may be key 7α-dehydroxylating bacteria in the human gut. The *baiE* gene abundance of cluster 1 was significantly and positively correlated with the ratio of secondary BAs to primary BAs. Furthermore, the *baiE* gene abundances of cluster 1 were significantly negatively correlated with inflammatory bowel disease, including Crohn’s disease and ulcerative colitis, as well as advanced nonalcoholic fatty liver disease, liver cirrhosis, and ankylosing spondylitis. Phylogenetic and metagenome-assembled genome analyses showed that the 7α-dehydroxylating bacterial clade of cluster 1 was affiliated with the family *Oscillospiraceae* and may demonstrate efficient BA dehydroxylation ability by harboring both a complete *bai* operon, for proteins which produce secondary BAs from primary BAs, and a gene for bile salt hydrolase, which deconjugates BAs, in the human gut.

**IMPORTANCE** In this study, we identified a key 7α-dehydroxylating bacterial group predicted to be largely responsible for converting primary bile acids (BAs) to secondary BAs in the human gut through sequence similarity network, genome neighborhood network, and gene abundance analyses using human gut metagenomes. The key bacterial group was phylogenetically quite different from known 7α-dehydroxylating bacteria, and their abundance was highly correlated with the occurrence of diverse diseases associated with bile acid 7α-dehydroxylation. In addition, we characterized the metabolic features of the key bacterial group using their metagenome-assembled genomes. This approach is useful to identify and characterize key gut bacteria highly associated with human health and diseases.

## INTRODUCTION

Bile acids (BAs), steroidal natural products synthesized from cholesterol in the liver, are secreted into the duodenum to solubilize lipids for digestion. BAs play a role in numerous biological processes, mostly related to physiological metabolism (1, 2). Primary BAs, such as cholic acid (CA) and chenodeoxycholic acid (CDCA), are initially secreted conjugated with either taurine or glycine but are then metabolized by deconjugation, dehydrogenation, dehydroxylation, and epimerization by the gut microbiome into other BAs, which have been shown to have an impact on human health (1, 3–6). For example, 7α-dehydroxylation of primary BAs such as CA and CDCA to secondary BAs such as deoxycholic acid (DCA) and lithocholic acid (LCA) greatly affects endocrine function and physiological metabolism, and the amount and balance of these compounds may influence human disease (2, 7–14).

Links between inflammatory bowel disease (IBD), including Crohn’s disease (CD) and ulcerative colitis (UC), and primary and secondary BAs have been repeatedly demonstrated (15–17). Liver cirrhosis (LC), liver cancer, colorectal cancer (CRC), nonalcoholic fatty liver disease (NAFLD), atherosclerotic cardiovascular disease (ACVD), ankylosing spondylitis (AS), type 2 diabetes mellitus (T2DM), and mitigation of the inflammatory response caused by Clostridioides difficile infection have also been associated with the amount and balance of primary and secondary BAs (11, 12, 16, 18–25). Additionally, secondary BAs can greatly influence the human gut microbial community owing to their antimicrobial ability—for example, having a strong inhibitory effect on Clostridioides difficile infections, a cause of antibiotic-associated diarrhea and colitis (14, 26). Therefore, a precise understanding of BA 7α-dehydroxylating bacteria converting the primary BAs CA and CDCA to the secondary BAs DCA and LCA, respectively, in the human gut and their relationship with human disease may help lead to the development of therapeutic options for BA-related diseases (27).

Several BA 7α-dehydroxylating bacterial species belonging to the genus *Clostridium* have been isolated and their BA metabolic pathways and bile acid-inducible (*bai*) operons well elucidated (1, 5, 28–36); however, biochemical mechanisms and the role of 7α-dehydroxylating bacteria in the human gut remains unclear. In addition, because the majority of intestinal bacteria are widely considered unculturable in the laboratory, many other medically important 7α-dehydroxylating bacteria in the human gut may remain uncultured or unexplored. Therefore, in this study, comprehensive bioinformatic analyses, including sequence similarity network (SSN), genome neighborhood network (GNN), phylogenetic tree, and relative abundance analysis, of BA 7α-dehydratase (BaiE), a key protein involved in the conversion of primary BAs to secondary BAs, were performed to investigate the diversity and human disease association of 7α-dehydroxylating bacteria in the human gut.

## RESULTS AND DISCUSSION

### Identification of putative BaiE sequences from human gut assembly metagenomes.

Experimentally verified BaiE sequences of Clostridium scindens VPI 12708 (32), *C. scindens* ATCC 35704 (33), Clostridium hylemonae DSM 15053 (29), and Clostridium hiranonis DSM 13275 (34) were used as initial query sequences for BLASTP searches for putative BaiE sequences (Table S1 in the supplemental material). To further analyze these sequences, we used SSN and GNN approaches. SSN analysis is a powerful approach to visualize relationships within large collections of proteins, while GNN analysis provides a higher level of information about the functional role of specific proteins because functionally associated genes are often organized as coexpressional functional networks in genomes. However, most human gut metagenomic contigs are too short to predict gene function by GNN analysis. Therefore, putative BaiE sequences in UniRef databases with completely clustered gene sets were also used as query sequences for searching BaiE sequences from human gut assembly metagenomes. Eleven putative BaiE sequences were identified from the UniRef100 database through repetitive BLASTP, SSN, and GNN analyses (Table S2 in the supplemental material).

10.1128/msystems.00455-22.3 TABLE S1 Bile acid 7α-dehydratase (BaiE) sequences used as the initial query sequences for BLASTP search in this study. They all were verified as BaiE experimentally. Download Table S1, PDF file, 0.1 MB. Copyright © 2022 Kim et al. 2022 Kim et al. https://creativecommons.org/licenses/by/4.0/ This content is distributed under the terms of the Creative Commons Attribution 4.0 International license.

10.1128/msystems.00455-22.4 TABLE S2 Putative BaiE sequences newly searched from the UniRef100 database. The BaiE protein sequences of Table S1 were also searched from the UniRef100 database. Download Table S2, PDF file, 0.1 MB. Copyright © 2022 Kim et al. 2022 Kim et al. https://creativecommons.org/licenses/by/4.0/ This content is distributed under the terms of the Creative Commons Attribution 4.0 International license.

**FIG 1:**
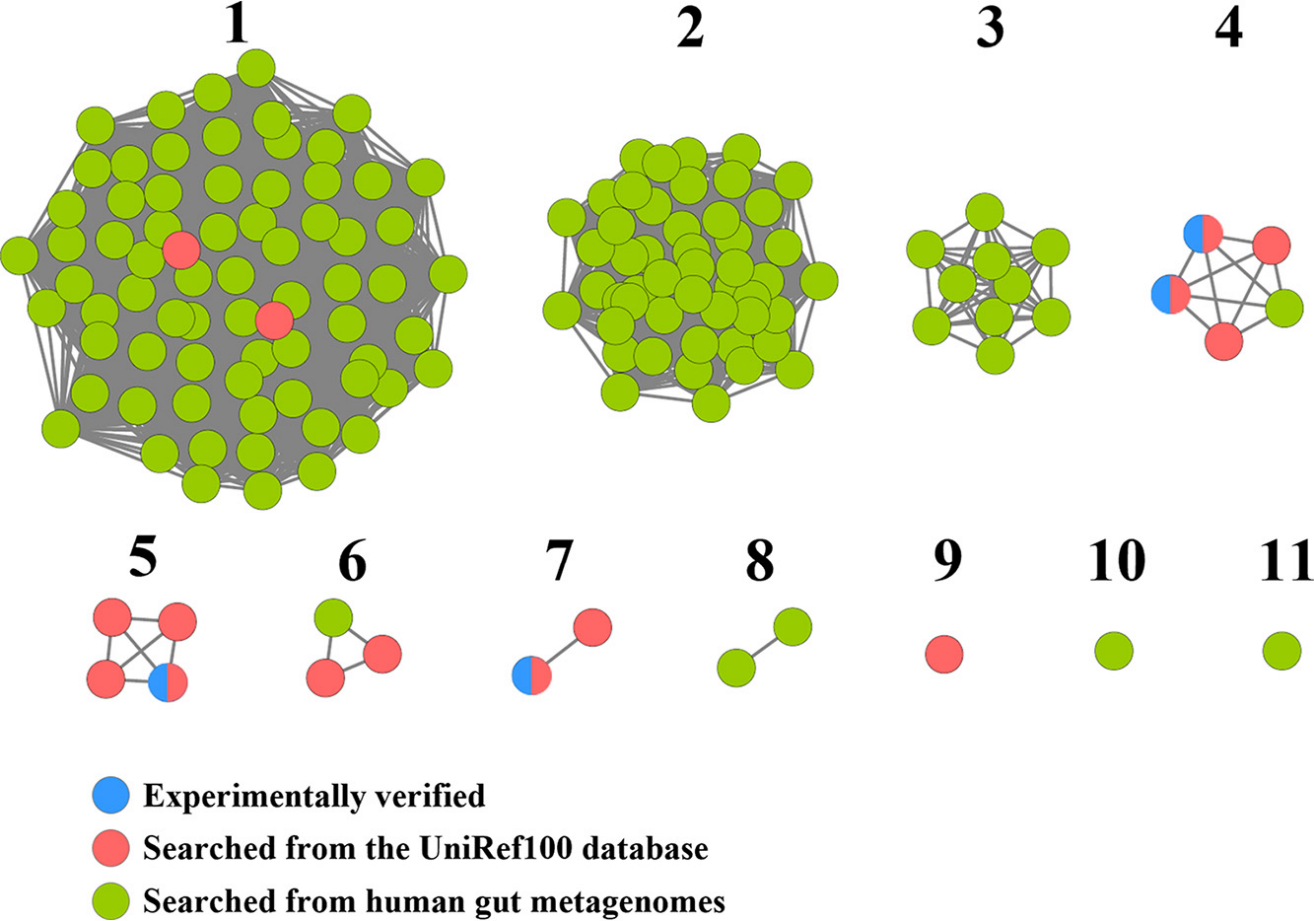
Sequence similarity networks (SSNs) of 135 protein sequences (green) identified from human gut microbiome whole-genome sequences, based on an alignment score of 70 (approximately 90% sequence identity). The BaiE sequences that had been previously experimentally verified (blue) or obtained from the UniRef100 database (red) were also used to generate the SSNs. Each node in the SSNs represents a protein, and alignment scores between proteins in different clusters are <70. Proteins within the same clusters have sequence identities of >92.4%. Numbers reflect arbitrary naming of clusters.

Whole-genome shotgun assembly sequences of human gut microbiomes in the Human Microbiome Project (HMP) portal were used for searching human gut BaiE sequences to identify a total of 135 protein sequences from 90 metagenome samples (Table S3 in the supplemental material). To investigate relationships among these proteins, an SSN for the 135 protein sequences and query sequences was constructed, and the proteins were separated into 11 clusters based on sequence identity (Fig. 1)—proteins within the same cluster had >92.4% sequence identity. Among 11 clusters, six clusters (clusters 1, 4, 5, 6, 7, and 9) included at least one query BaiE sequence, suggesting that proteins in these six clusters may have BaiE activity. In particular, cluster 1, comprising the largest number of protein sequences, included two putative BaiE query sequences derived from the UniRef100 database, which suggests that proteins of cluster 1 may be the most abundant BaiE in the human gut. Proteins of five clusters (clusters 2, 3, 8, 10, and 11) that did not contain query BaiE sequences required additional GNN analysis to infer their functions.

10.1128/msystems.00455-22.5 TABLE S3 GenBank SRA accession numbers of human gut metagenomes in which putative BaiE protein sequences were identified. Download Table S3, PDF file, 0.1 MB. Copyright © 2022 Kim et al. 2022 Kim et al. https://creativecommons.org/licenses/by/4.0/ This content is distributed under the terms of the Creative Commons Attribution 4.0 International license.

### Phylogenetic analysis of putative BaiE proteins and GNN analysis of *baiE* genes.

**FIG 2:**
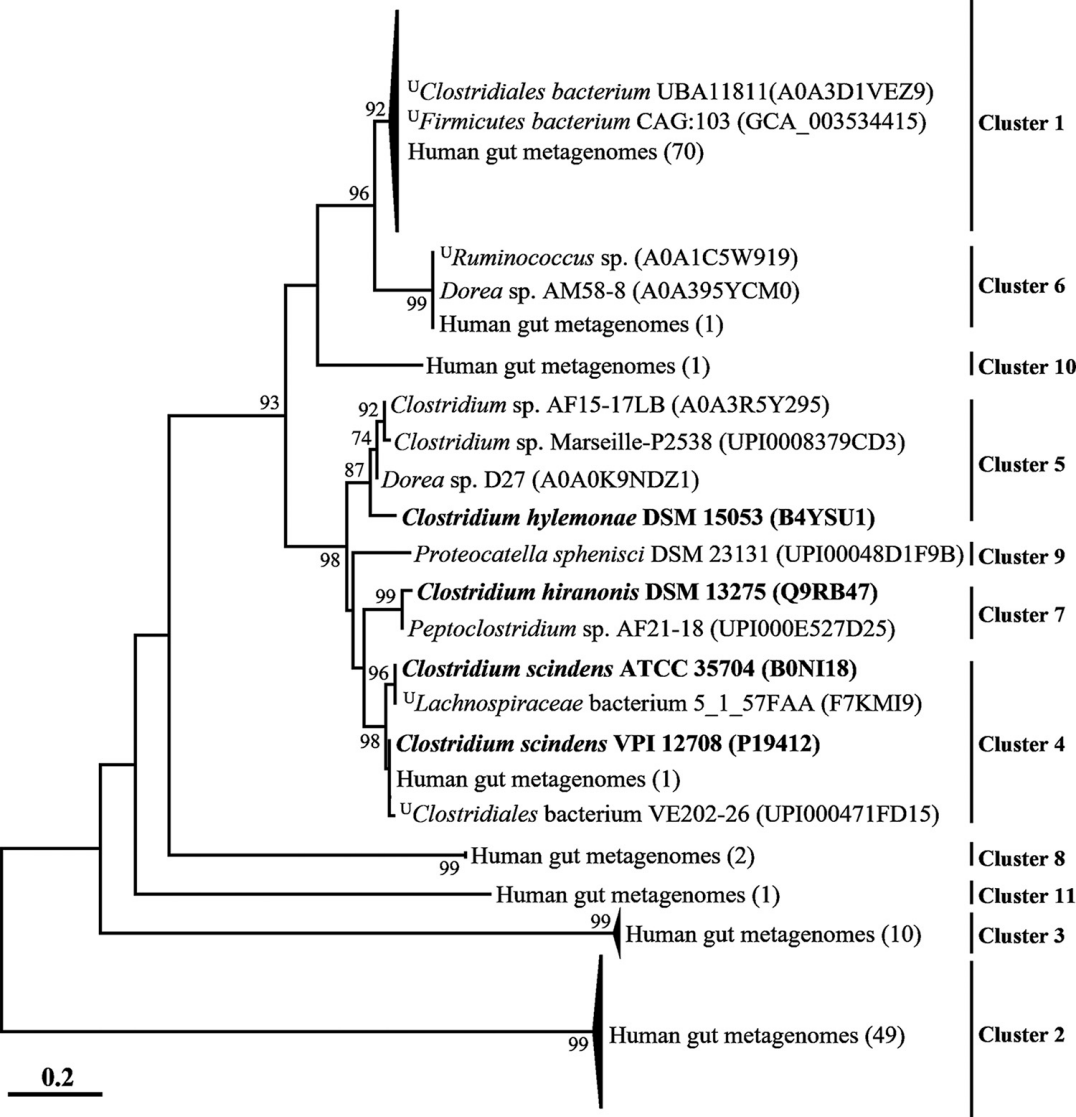
Phylogenetic tree of putative BaiE sequences based on the maximum-likelihood algorithm. Names of strains harboring putative BaiE sequences are in parentheses, and the superscript “U” indicates uncultured strains. Organisms whose BaiE sequences were previously experimentally verified are in bold. Bootstrap values of >70% are shown on the branches as percentages of 1,000 replicates.

To obtain a detailed view of the phylogenetic relationships across the BaiE clusters, a phylogenetic analysis was performed (Fig. 2). Protein sequences that grouped into different clusters (Fig. 1) were also well separated, with very high bootstrap values in the phylogenetic analysis, and proteins from the same clusters were always tightly clustered together. Clusters 1, 4, 5, 6, 7, and 9, which included query BaiE sequences, clustered together, but clusters 2, 3, 8, and 11 (which did not include query BaiE sequences) formed distant phylogenetic lineages. Protein sequences of cluster 1, comprising the largest number of protein sequences, were tightly clustered with putative BaiE sequences of uncultured *Firmicutes* bacterium CAG:103 and uncultured *Clostridiales* bacterium UBA11811, available in the UniRef100 database, with very high sequence identities (>97.2%). These results suggest that bacterial groups harboring protein sequences of cluster 1 may be closely related and abundant 7α-dehydroxylating bacteria in the human gut. Although metagenome-assembled genomes (MAGs) harboring *baiE* genes of cluster 1 are currently available, bacterial strains harboring *baiE* genes of cluster 1 have not been cultivated to date, suggesting that they are probably difficult to culture under general laboratory culture conditions. Some MAGs and isolates, namely, *Firmicutes* bacterium CAG:103 in cluster 1 and *Dorea* sp. AM58-8 in cluster 6, were reported as BA 7α-dehydroxylating candidates possibly producing secondary BAs from primary BAs (37). Specifically, the literature has reported that the bacterial clade including *Firmicutes* bacterium CAG:103 may be abundantly present as *Ruminococcaceae* family members in the human gut. These results suggest that bacterial clades harboring *baiE* genes of cluster 1 may be primarily responsible for producing secondary BAs from primary BAs in the human gut.

**FIG 3:**
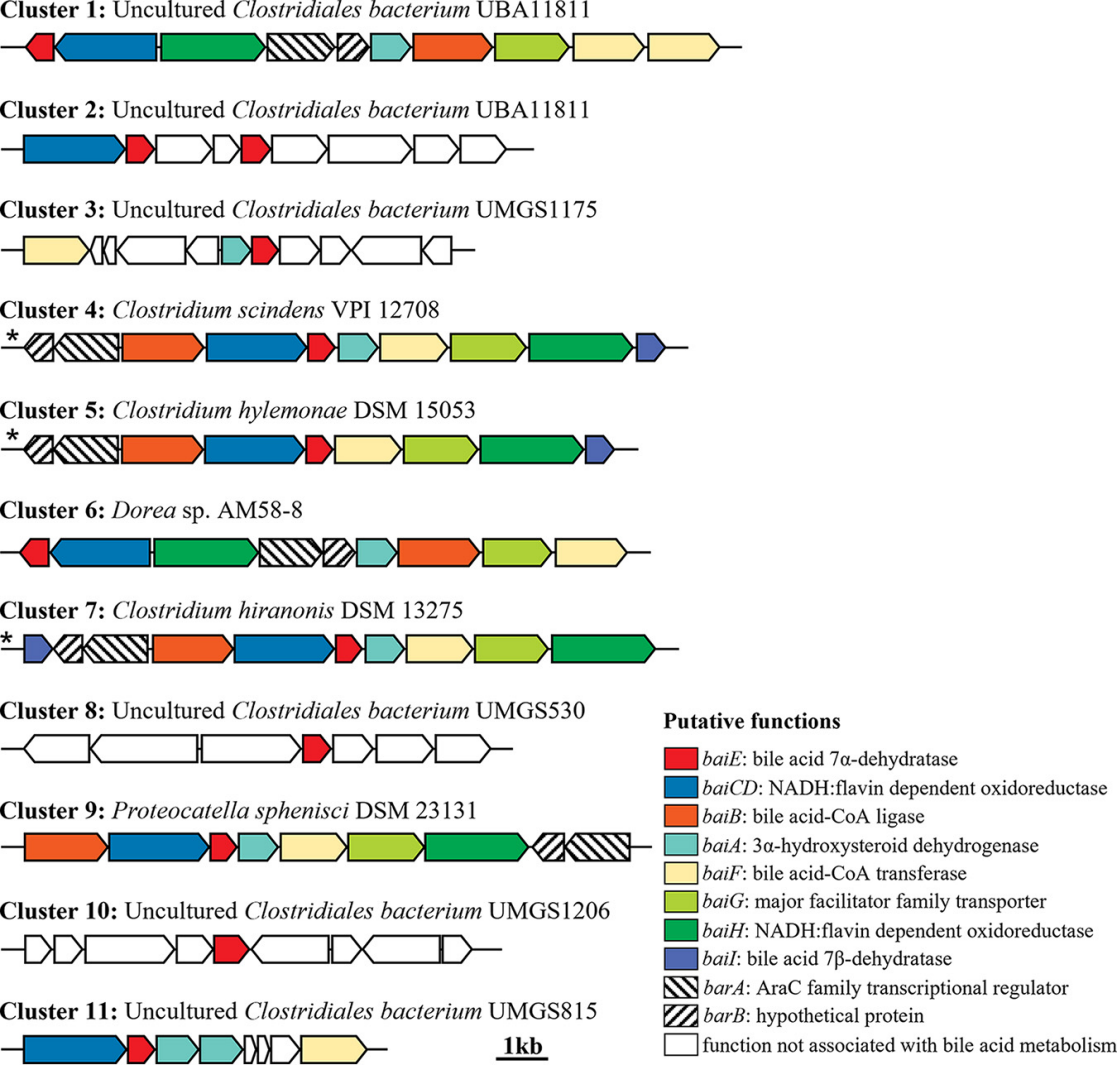
Gene neighborhood analysis of putative *baiE* genes present in the *bai* operons of representative genomes of each phylogenetic cluster (Fig. 2). Asterisks indicate gene structures of *bai* operons that were previously experimentally verified to be able to convert primary bile acids to secondary bile acids.

BaiE is essential for the conversion of primary BAs to secondary BAs via a 7α-dehydroxylation. However, besides BaiE, the 7α-dehydroxylation of primary BAs is accomplished via multistep biochemical reactions by several enzymes, including those which promote the uptake of BAs (BaiG, major facilitator family transporter), conjugation to coenzyme A (CoA) (BaiB, bile acid-CoA ligase), dehydrogenation (BaiA, 3α-hydroxysteroid dehydrogenase), oxidation (BaiCD and BaiH, NADH:flavin oxidoreductase), and hydrolysis of CoA (BaiF, bile acid-CoA transferase), encoded in the BA-inducible (*bai*) operon (5, 30, 34–36). Proteins responsible for reduction of BAs (BaiN, bile acid 5β-reductase) and hydrolysis of CoA (BaiK, bile acid-CoA transferase) encoded distantly from the *bai* operon are also considered necessary for the 7α-dehydroxylation of primary BAs (30, 35). Therefore, a GNN analysis of putative *baiE* genes aimed to provide more definite functional information. GNN analysis showed that *bai* operons in the genomes or MAGs of clusters 1, 4, 5, 6, 7, and 9 harbored all the genes required for the 7α-dehydroxylation of primary BAs (Fig. 3), suggesting that bacteria belonging to clusters 1, 4, 5, 6, 7, and 9 can be BA 7α-dehydroxylating candidates to produce secondary BAs from primary BAs. However, because the operons of clusters 2, 3, 8, 10, and 11 did not harbor the complete gene set required to convert primary BAs to secondary BAs, bacteria belonging to clusters 2, 3, 8, 10, and 11 may not be BA 7α-dehydroxylating candidates. MAGs of cluster 1, comprising the largest number of BaiE protein sequences, harbored all genes identified from the *bai* operons of strains already experimentally verified in clusters 4, 5, and 7, but their operon structures were slightly different. Characteristics of each gene in the *bai* operon of cluster 1 are summarized in Table 1.

**TABLE 1:** Metabolic genes of a putative *baiE* operon identified from the genome of the uncultured *Clostridiales* bacterium UBA11811 of cluster 1

GenBank protein ID	Putative function and gene	Length (aa)^*a*^	Most similar gene product(s) in GenBank (species, accession no.)	Identity (%)
HCG31418	Bile acid 7α-dehydratase, *baiE*	177 (−)	Nuclear transport factor 2 family protein (*Dorea* sp. AM58-8, RGY81629)	83.3
HCG31419	NADH:flavin dependent oxidoreductase, *baiCD*	644 (−)	NADH oxidase (uncultured *Ruminococcus* sp. strain 2789STDY5608817, SCI19301)	76.1
HCG31420	NADH:flavin dependent oxidoreductase, *baiH*	663 (+)	NADH oxidase (uncultured *Ruminococcus* sp. strain 2789STDY5608817, SCI19270)	82.7
HCG31421	AraC family transcriptional regulator, *barA*	514 (+)	l-Rhamnose operon transcriptional activator RhaR (uncultured *Eubacterium* sp. strain 2789STDY5834872, SCH90625)	67.9
HCG31422	Hypothetical protein, *barB*	195 (+)	Uncharacterized protein (uncultured *Eubacterium* sp. strain 2789STDY5834872, SCH90593)	55.3
HCG31423	Bile acid 7-dehydroxylase, *baiA*	255 (+)	SDR family NAD(P)-dependent oxidoreductase (*Dorea* sp. AF36-15AT, RHP08808)	61.1
HCG31424	Bile acid-CoA ligase, *baiB*	506 (+)	Long-chain-fatty-acid–CoA ligase (uncultured *Eubacterium* sp. strain 2789STDY5834872, SCH90517)	74.8
HCG31425	Major facilitator family transporter, *baiG*	473 (+)	Bile acid transporter (Clostridium hiranonis TO-931, AAF22849)	47.7
HCG31426	Bile acid-CoA hydrolase, *baiF*	452 (+)	CoA transferase (*Dorea* sp. AM58-8, RGY81637)	90.0
HCG31427	Bile acid-CoA hydrolase, *baiF*	478 (+)	Bile acid-CoA hydrolase (uncultured *Eubacterium* sp. strain 2789STDY5834872, SCH90440)	71.3

a aa, amino acids. The orientation of the coding strand is indicated in parentheses.

### Conversion of cholate by BaiA and BaiB enzymes.

**FIG 4:**
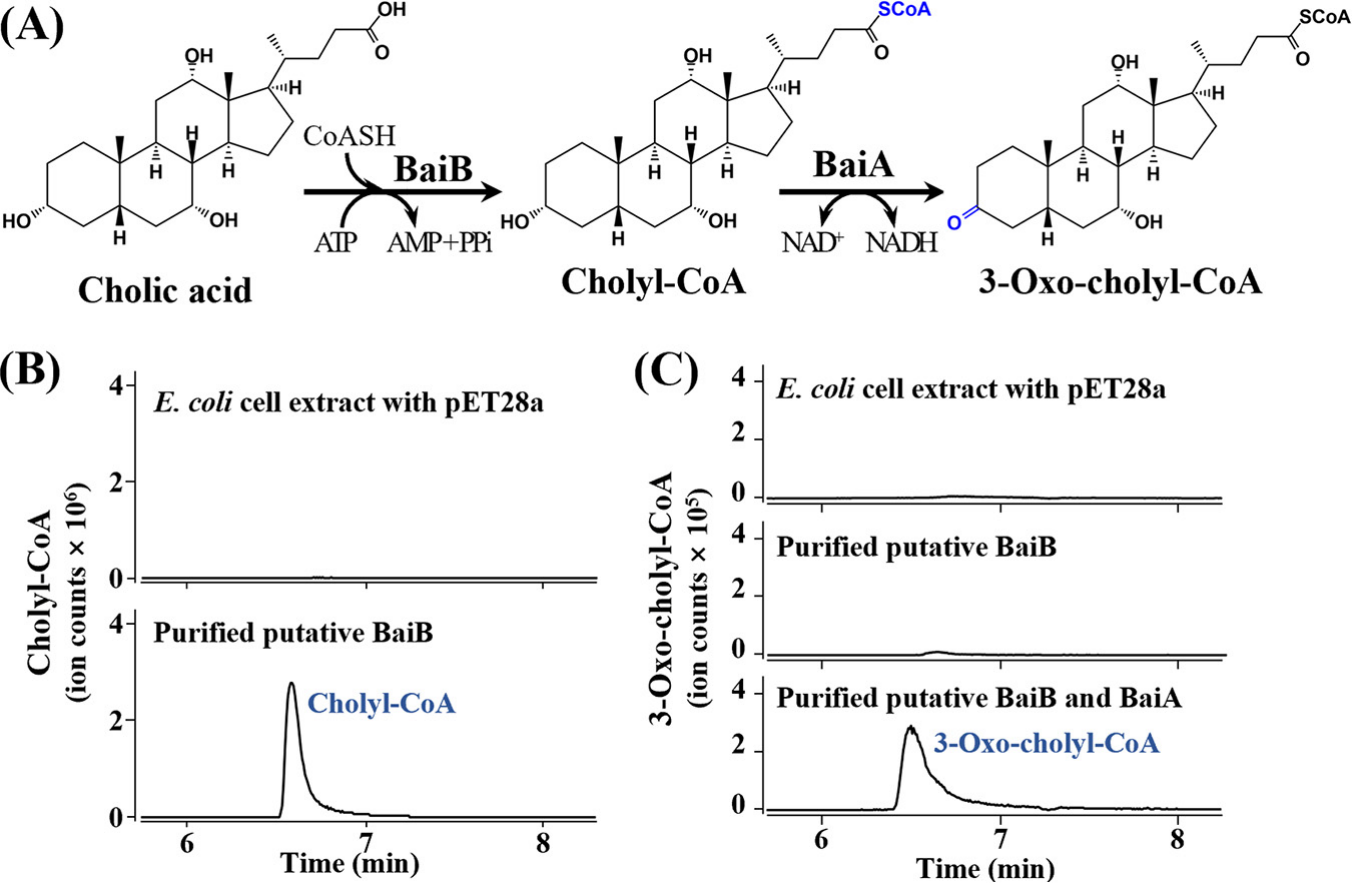
Confirmation of enzymatic functions of the putative *baiA* (DER43_01330) and *baiB* (DER43_01335) genes in the uncultured *Clostridiales* bacterium UBA11811. A diagram showing the enzymatic reactions of cholic acid by BaiA and BaiB proteins (A). LC-Q-TOF-MS ion chromatograms showing the production of cholyl-CoA (*m/z* 1158.4026 of [M+H]^+^, 6.544 min) by BaiB (B) and 3-oxo-cholyl-CoA (*m/z* 1156.3852 of [M+H]^+^, 6.428 min) by BaiB and BaiA (C) from cholic acid. Cell lysate of E. coli BL21(DE3) harboring the pET28a plasmid without a DNA insert was used as a negative control.

To confirm whether the *bai* operon in cluster 1 is responsible for BA 7α-dehydroxylation, the putative *baiA* and *baiB* genes, which might be involved in upstream reactions of the BA 7α-dehydroxylation pathway in uncultured *Clostridiales* bacterium UBA11811 of cluster 1, were overexpressed (Fig. S2), and their functions were confirmed (Fig. 4). The purified BaiB protein clearly exhibited CoA ligation activity to produce cholyl-CoA from CA (Fig. 4B), suggesting that the putative *baiB* gene is a bile acid-CoA ligase gene that ligates CoA to CA or CDCA. In addition, purified BaiB and BaiA protein mixtures were shown to produce 3-oxo-cholyl-CoA from CA, but no production of 3-oxo-cholyl-CoA by only the purified BaiB protein was observed (Fig. 4C), suggesting that the BaiA protein is a bile acid dehydroxylase that dehydroxylates cholyl-CoA or chenodeoxycholyl-CoA. The conversion of CA to cholyl-CoA and 3-oxo-cholyl-CoA by BaiB and BaiA proteins suggests that the *bai* operons in cluster 1 may be responsible for BA 7α-dehydroxylation to produce secondary BAs from primary BAs in the human gut (38, 39).

10.1128/msystems.00455-22.1 FIG S1 Relative *baiE* gene abundances of BaiE clusters in mouse gut microbiota. The abundance of *baiE* genes in each sample was normalized based on read counts per million sequencing reads. Blue circles indicate the mean values of the relative abundances of *baiE* genes in each cluster. Mean values, with percentages of total counts in parentheses, are also indicated above the data. Download FIG S1, PDF file, 0.1 MB. Copyright © 2022 Kim et al. 2022 Kim et al. https://creativecommons.org/licenses/by/4.0/ This content is distributed under the terms of the Creative Commons Attribution 4.0 International license.

10.1128/msystems.00455-22.2 FIG S2 SDS-PAGE analysis and Coomassie blue staining of the proteins expressed by IPTG-induced E. coli BL21(DE3) cells harboring the pET28a-*baiB* (DER43_01335) (A) and pET28a-*baiA* (DER43_01330) (B) constructs. Lanes M, protein molecular mass marker (GeneDireX, USA); 1, cell extract pellet; 2, cell extract supernatant; 3, fraction eluted using elution buffer with 300 mM imidazole. Download FIG S2, PDF file, 0.1 MB. Copyright © 2022 Kim et al. 2022 Kim et al. https://creativecommons.org/licenses/by/4.0/ This content is distributed under the terms of the Creative Commons Attribution 4.0 International license.

### Relative abundance of *baiE* genes and *baiE* gene transcripts of BaiE clusters in human gut microbiota.

**FIG 5:**
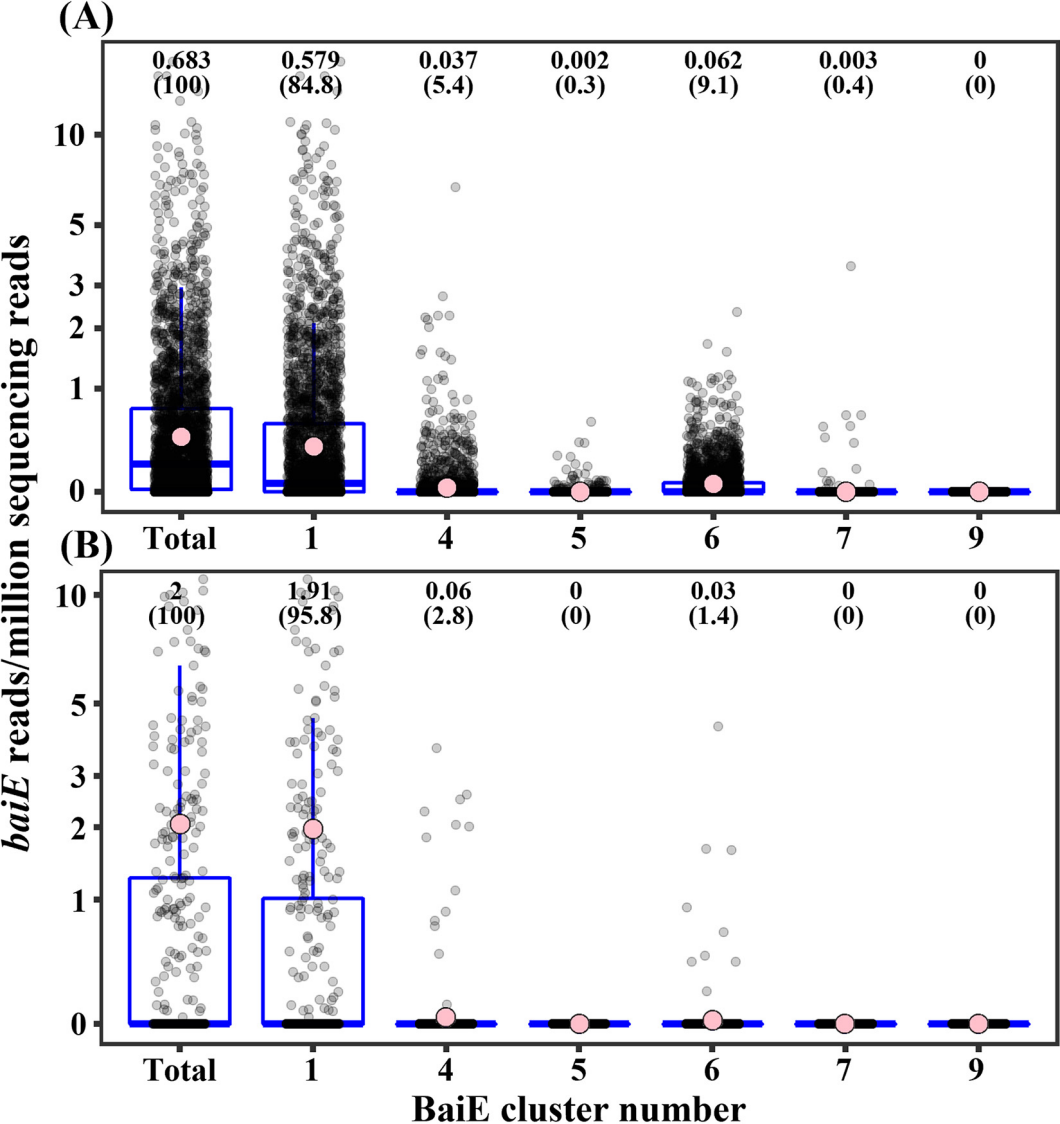
Relative abundances of *baiE* genes (A) and *baiE* gene transcripts (B) of BaiE clusters in the human gut microbiota. The abundance of *baiE* genes and *baiE* gene transcripts in each sample was normalized based on read counts per million sequencing reads. Pink circles indicate the mean values of the relative abundances of *baiE* genes or *baiE* gene transcripts in each cluster. Mean values, with percentages of total counts in parentheses, are also indicated above the data.

The relative abundance of the *baiE* genes of putative BaiE clusters 1, 4, 5, 6, 7, and 9 was analyzed to investigate which BaiE clusters are majorly responsible for the conversion of primary BAs to secondary BAs in the human gut. *baiE* genes of cluster 1 were predominantly abundant and comprised nearly 85% of total *baiE* genes in the human gut microbiota (Fig. 5A). *baiE* genes of cluster 6, which included *Dorea* sp. AM58-8, which was not experimentally verified as a 7α-dehydroxylating bacterium, were the next most abundant, at approximately 9.1% relative abundance of total *baiE* genes. In contrast, *baiE* genes of clusters 4, 5, and 7, which included experimentally verified BaiE sequences, had low relative abundances of only 5.4%, 0.3%, and 0.4%, respectively, suggesting that they may not be major 7α-dehydroxylation bacteria in the human gut. *baiE* genes of cluster 9, including a putative *baiE* gene sequence from MAGs, was identified at a very low abundance (~0%). The metatranscriptome analysis of human gut microbiota showed that *baiE* gene transcripts of cluster 1 were predominantly identified and comprised approximately 95.8% of total *baiE* gene transcripts, while those of cluster 4, which included *C. scindens*, experimentally verified as a 7α-dehydroxylating bacterium, and cluster 6, with the second highest abundance of *baiE* genes, were only 2.8% and 1.4%, respectively (Fig. 5B). *baiE* gene transcripts of other BaiE clusters were not identified. The abundance analysis of *baiE* genes and *baiE* gene transcripts in the human gut microbiota clearly suggests that bacterial groups harboring *baiE* genes of cluster 1 may have a greater role in converting primary BAs to secondary BAs in the human gut than those in the other clusters.

Many previous studies have suggested that the balance between primary BAs and secondary BAs or the amount of BAs present in the gut may be closely associated with IBD (15, 17, 40), NAFLD (20, 41, 42), LC (18), liver cancer (11), CRC (16, 43), and ACVD (22, 44). Therefore, correlations between the relative *baiE* gene abundance of each cluster and above-mentioned diseases were investigated using the public gut metagenome cohort data. However, because gut metagenome data of patients with liver cancer were not available, the correlation between the relative *baiE* gene abundance and liver cancer was not investigated. The analysis showed that relative *baiE* gene abundances of clusters 1, 4, and 6, which have high *baiE* gene abundance in the human gut, were significantly correlated with several human diseases (Fig. 5), while those of clusters 5, 7, and 9, which have low *baiE* gene abundance, did not show significant correlation with any of the above-mentioned diseases (data not shown).

**FIG 6:**
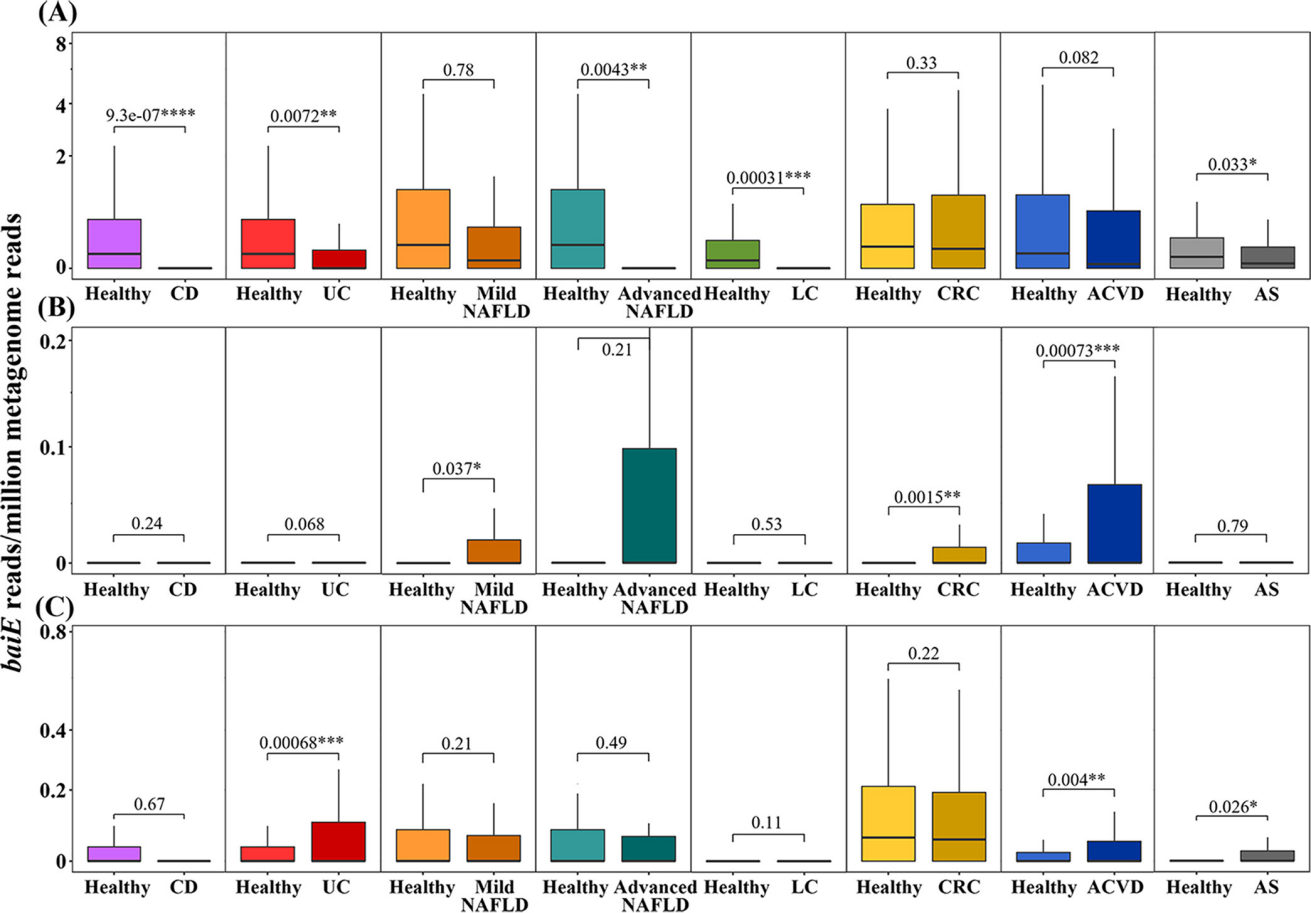
Relative abundance of *baiE* genes of BaiE clusters 1 (A), 4 (B), and 6 (C) according to disease cases in human gut metagenomes. *, *P < *0.05; **, *P < *0.01; ***, *P < *0.001. CD, Crohn’s disease; UC, ulcerative colitis; NAFLD, nonalcoholic fatty liver disease; LC, liver cirrhosis; CRC, colorectal cancer; ACVD, atherosclerotic cardiovascular disease; AS, ankylosing spondylitis.

It has been continually demonstrated that increasing the level of secondary BAs in the gut suppresses the occurrence and development of IBD, including CD and UC, because secondary BAs have a strong inhibitory effect on gut inflammation by repressing the synthesis of proinflammatory cytokines (12, 15, 40, 45). Therefore, it is expected that the abundance of 7α-dehydroxylation bacteria would be negatively correlated with CD and UC occurrences. In agreement with these findings, the relative *baiE* gene abundance of cluster 1 was significantly higher in healthy people than in patients with CD or UC (Fig. 6A). However, relative *baiE* gene abundances of clusters 4 and 6 were very low and were not significantly correlated with CD or UC. The relative *baiE* gene abundance of cluster 4, which includes a well-verified *baiE* gene of *C. scindens*, was very low and had no significant correlation with CD or UC occurrence (Fig. 6B). Moreover, the relative *baiE* gene abundance of cluster 6 was significantly higher in patients with UC than in healthy people (*P < *0.001), in disagreement with previous findings (Fig. 6C), suggesting that other factors besides the formation of secondary BAs by 7α-dehydroxylating bacteria of cluster 6 may contribute more to the occurrence of UC in the human gut. These results suggest that bacterial groups harboring *baiE* genes of cluster 1 may be the most important 7α-dehydroxylating bacteria associated with the occurrence of IBD in the human gut.

It has been reported that small intestinal bacterial overgrowth and gut microbial dysbiosis (46, 47) as well as an increase in total BAs, especially secondary BAs (42, 48), are more frequently observed in patients with NAFLD than in healthy people. Previous studies have proposed a BA-associated intervention model for NAFLD occurrence by using gut microbial dysbiosis: the decreased stimulation of farnesoid X receptor (FXR), which regulates lipid metabolism, by an increased ratio of secondary BAs (DCA and LCA) to primary BAs (CA and CDCA) caused by gut microbial dysbiosis, leading to primary BA synthesis, glucose metabolism impairment, and dysregulation of lipid homeostasis in hepatocytes, eventually causing hepatic steatosis (13, 20, 49). This BA-associated NAFLD development model suggests that the abundance of BA 7α-dehydroxylating bacteria in the human gut may be positively correlated with NAFLD occurrence. However, the relative *baiE* gene abundance of cluster 1, the major *baiE* gene cluster in the human gut, was higher in healthy people than in patients with advanced NAFLD (Fig. 6A), suggesting that secondary BAs may not be associated with NAFLD causality or that BA 7α-dehydroxylating bacteria of BaiE cluster 1 may suppress the occurrence or development of advanced NAFLD. On the contrary, the relative *baiE* gene abundance of BaiE cluster 4 was higher in patients with NAFLD (Fig. 6B), and *baiE* gene abundance of cluster 6 had no significant correlation with NAFLD (Fig. 6C). It is therefore unclear if an increase in secondary BAs is causative for NAFLD.

LC is an advanced irreversible liver disease caused by acute or chronic liver injury due to alcohol abuse, liver toxins, hepatitis virus infection, or immune dysfunction (primary biliary cholangitis or primary sclerosing cholangitis) (13). Bajaj et al. (50) reported that gut microbial dysbiosis may be associated with the occurrence of LC. The relative *baiE* gene abundance of cluster 1 was significantly higher in healthy people than in patients with LC (*P < *0.001) (Fig. 6A). However, relative *baiE* gene abundances of clusters 4 and 6 had no significant correlation with LC (Fig. 6B and C). Kakiyama et al. (51) and Ridlon et al. (18) reported that BA concentrations, the ratio of secondary BAs to primary BAs, and BA 7α-dehydroxylating bacteria decreased in patients with LC because BA pool size is significantly decreased in damaged liver (cirrhosis state). These results suggest that the low *baiE* gene abundance of cluster 1 in patients with LC may not be associated with LC causality but may instead be caused by a decrease in BA production ability in patients with LC.

Secondary BAs influence several signaling pathways related to the generation of reactive oxygen and nitrogen species, disruption of cell membranes and mitochondria, and induction of DNA damage and apoptosis in enterocytes that can lead to the development of CRC (16, 43, 52), suggesting that the abundance of BA 7α-dehydroxylating bacteria in gut may be correlated with CRC occurrence (23). The relative *baiE* gene abundance of cluster 4, including the *C. scindens baiE* gene, was also significantly higher in patients with CRC than in healthy people (*P < *0.005) (Fig. 6B), although their abundance was very low. However, the relative *baiE* abundance of clusters 1 and 6, which showed high abundance, was not significantly correlated with CRC (Fig. 6A and C). These results suggest that other factors besides abundance of secondary BAs may influence the occurrence of CRC.

Several cohort studies have demonstrated that a shift of primary BAs to secondary BAs is observed in patients with heart failures such as ACVD (19, 22), suggesting that BA 7α-dehydroxylating bacteria in the human gut can be important causative agents of cardiovascular disease. Relative *baiE* gene abundances of clusters 4 and 6 in the human gut were also significantly higher in patients with ACVD than in healthy people (Fig. 6B and C), which was in accordance with a report that the abundance of *C. scindens* gut bacteria was higher in patients with ACVD than in healthy people (53). However, the relative *baiE* abundance of cluster 1 was higher in healthy people than in patients with ACVD, although this finding was not statistically significant (*P = *0.082). These results suggest that further studies on effects of cluster 1 BA 7α-dehydroxylating bacteria on the occurrence of ACVD may be necessary.

AS is a systemic, chronic, and inflammatory autoimmune disease characterized by the inflammation of the axial skeleton, peripheral joints, and ligament entheses. It was reported that the relative abundance of *Clostridium* and an unclassified *Lachnospiraceae* bacterium, inferred to be BA 7α-dehydroxylating bacteria, was lower in patients with AS than in healthy people (54), suggesting that the abundance of BA 7α-dehydroxylating bacteria may be positively correlated with the occurrence and development of AS. In fact, the relative *baiE* gene abundance of cluster 6 was significantly higher in patients with AS than in healthy people (Fig. 5C). However, the relative abundance of *baiE* from cluster 1 was significantly higher in healthy people than in patients with AS. These results suggest that further studies on effects of BA 7α-dehydroxylating bacteria on AS may be necessary. The association of BAs with the regulation of glycemic responses in T2DM has been suggested (12, 55), but no significant correlations between the relative *baiE* gene abundance of all clusters and T2DM were observed (data not shown).

### Correlations between *baiE* gene abundance and bile acid level.

**FIG 7:**
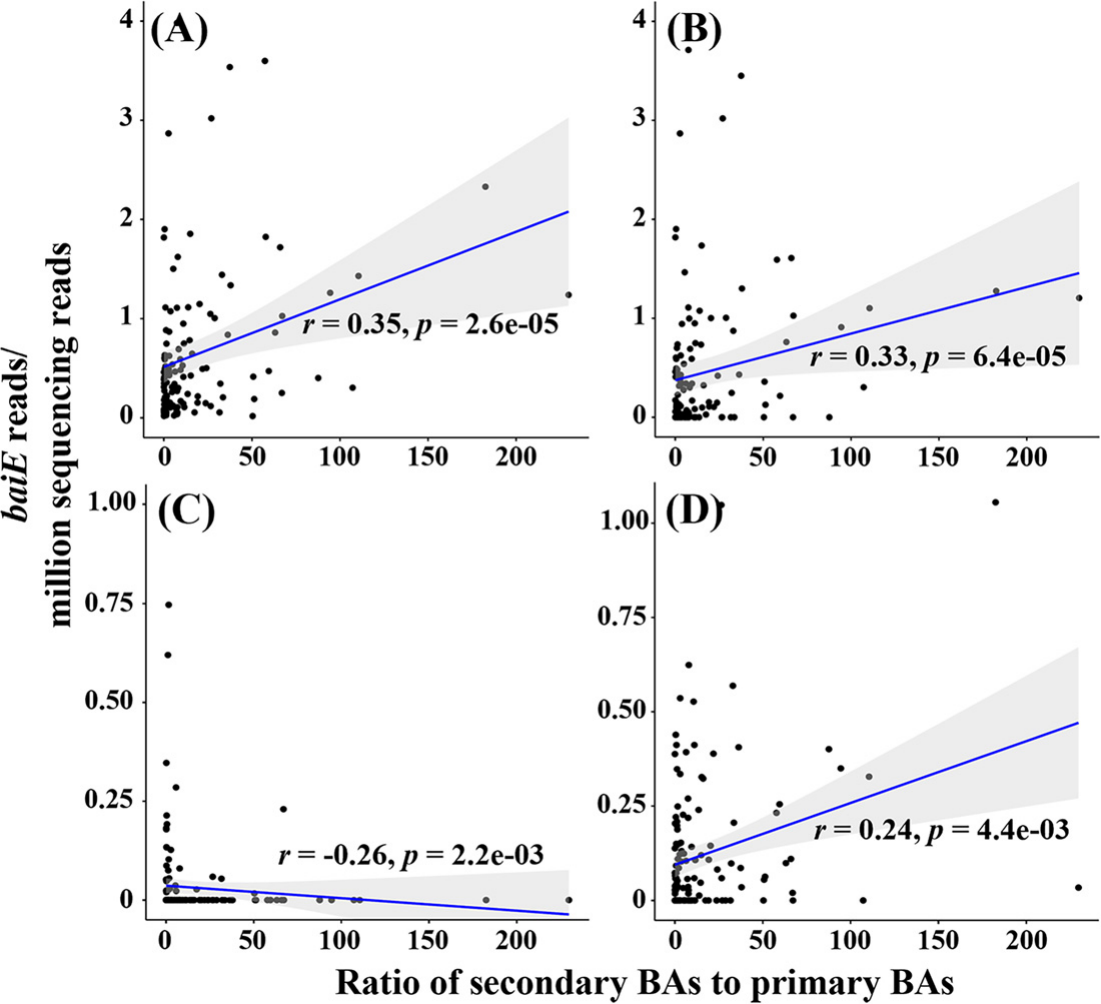
Spearman correlations between *baiE* abundances of all sequences (A), cluster 1 (B), cluster 4 (C), and cluster 6 (D) and ratios of secondary BAs to primary BAs in the human gut. Each dot represents the results from a participant. The *r* and *p* values are the Spearman correlation and significant coefficients, respectively.

Correlations between *baiE* gene abundance and bile acid levels (the ratios of secondary BAs to primary BAs) in the human gut were investigated. The results showed that the total abundance of all *baiE* genes identified in this study was significantly positively correlated with the ratio of secondary BAs to primary BAs (*r *= 0.35, *P = *2.6e−05) (Fig. 7A). Particularly notable is that the abundance of *baiE* genes in cluster 1 was significantly and positively (*r *= 0.33, *P = *6.4e−05) correlated with the ratio of secondary BAs to primary BAs (Fig. 7B), indicating that the bacterial clade harboring the *baiE* genes of cluster 1 may be majorly responsible for converting primary BAs to secondary BAs. The abundance of *baiE* genes in cluster 6 was also positively correlated with the ratio of secondary BAs to primary BAs with relatively great significance (*r *= 0.24, *P = *4.4e−03) (Fig. 7D). However, the abundance of *baiE* genes in cluster 4, including Clostridium scindens, was negatively correlated with the ratio of secondary BAs to primary BAs (*r* = −0.26, *P = *2.2e−03) (Fig. 7C), suggesting that the bacterial clade harboring the *baiE* genes of cluster 4 may not play an important role in converting primary BAs to secondary BAs in the human gut, which might well represent the results showing correlation between the *baiE* gene abundances of cluster 4 and disease occurrences in Fig. 6B.

### Phylogenetic and genomic features of BA 7α-dehydroxylating bacteria of cluster 1.

**FIG 8:**
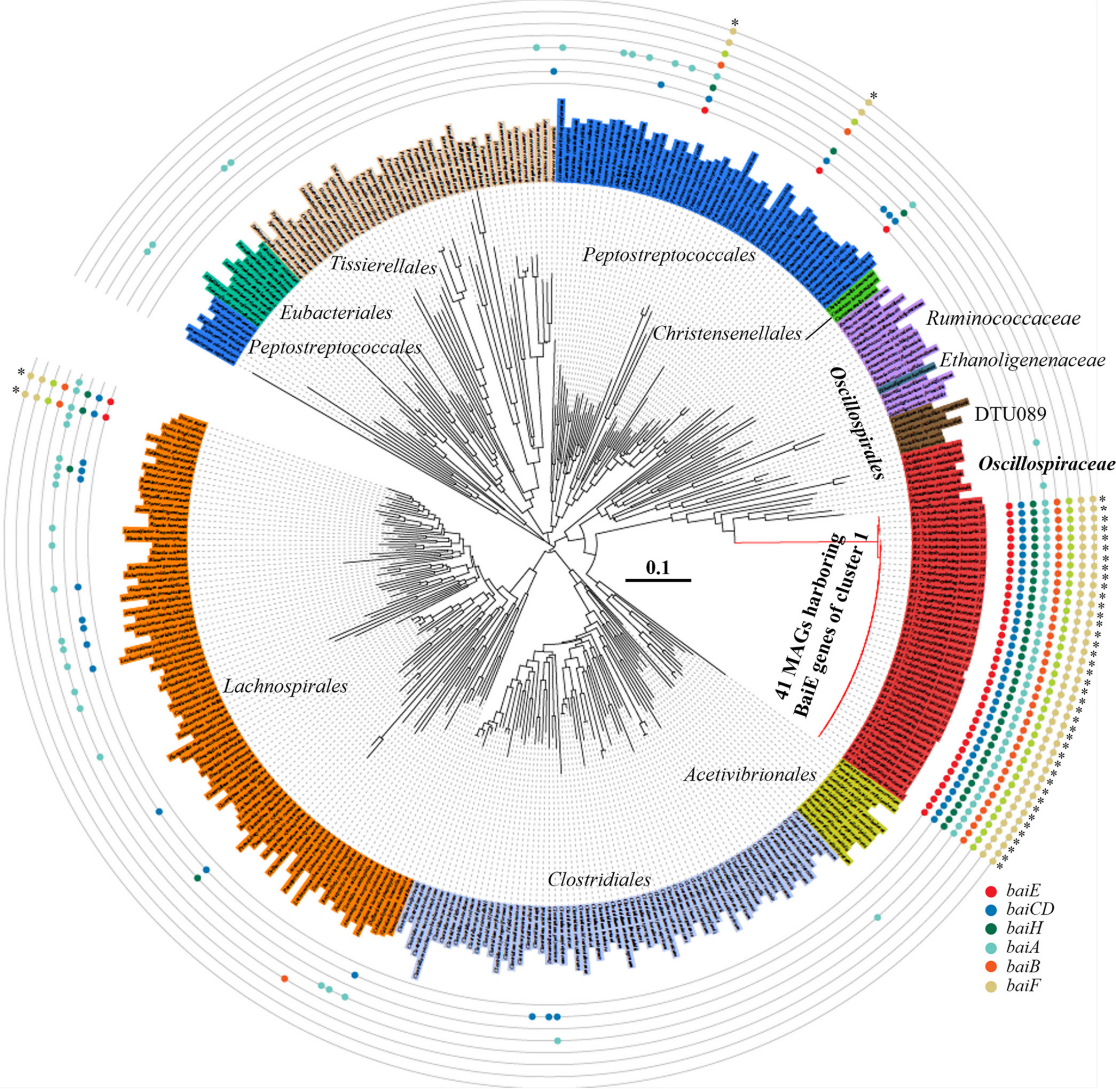
Neighbor-joining tree based on 120 concatenated marker proteins, showing the phylogenetic positions of 41 MAGs harboring BaiE genes of cluster 1. The tree consists of genomes of all cultured type strains belonging to the class *Clostridia*, except for the 41 MAGs. Colored circles indicate the possible presence of genes associated with 7α-dehydroxylation of primary BAs, and asterisks indicate genomes capable of converting primary BAs to secondary BAs by harboring a *bai* operon. The bar indicates the number of substitutions per site.

A total of 41 MAGs containing *baiE* genes of cluster 1 were identified from human gut MAG databases. These MAGs showed high BaiE protein sequence similarities (≥99.03%), suggesting that they were probably derived from similar phylogenomic lineages. Phylogenetic analysis using concatenated protein sequences of 120 bacterial marker genes revealed that all 41 MAGs formed a very close phylogenetic lineage with each other (Fig. 8). In addition, average nucleotide identity (ANI) values among the 41 MAGs were greater than 98.01%, higher than the 94 to 96% cutoff value that is generally accepted for species delineation (56), which suggests that the bacteria are identical at the species level. A phylogenetic tree indicated that 41 MAGs were clustered with type strains of the family *Oscillospiraceae* within the order *Eubacteriales*, indicating that they may be members of this family. However, the 41 MAGs also formed a phylogenetic lineage quite distant from other cultured *Oscillospiraceae* members, and ANI values between them and the genomes of cultured *Oscillospiraceae* type strains were below 68.2%, suggesting that the bacteria may have quite different genomic, metabolic, and physiological features from those of cultured *Oscillospiraceae* strains. The average genome size, total gene number, and G+C content of 41 MAGs were 1.88 ± 0.07 Mb, 1,785 ± 85, and 62.0 ± 0.3 mol%, respectively. All 41 MAGs harbored a complete *bai* operon, indicating their 7α-dehydroxylation function, and their *bai* operon structures were identical (Fig. 3). Except for 41 MAGs, only four cultured type strains (*C. scindens* ATCC 35704, *C. hylemonae* DSM 15053, *C. hiranonis* DSM 13275, and Proteocatella sphenisci DSM 23131) in the class *Clostridia* harbored a *bai* operon, and those operons were phylogenetically quite distant from MAG bacteria of cluster 1.

**FIG 9:**
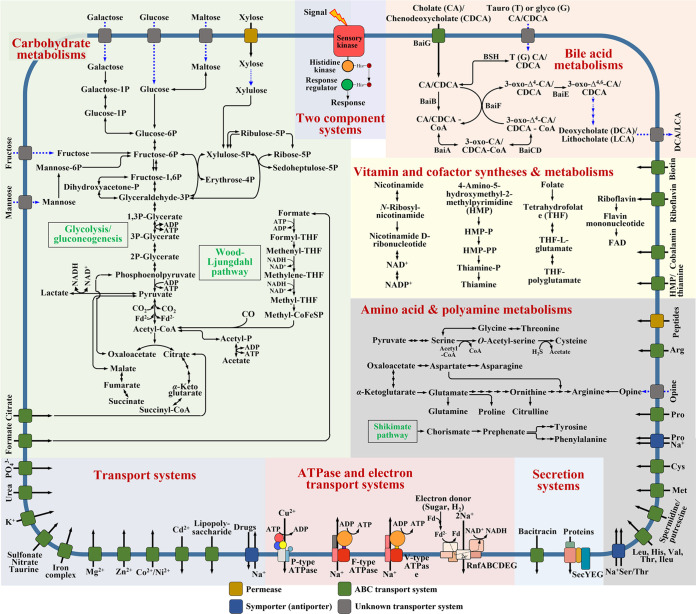
Proposed metabolic pathways for carbohydrates, bile acids, vitamins, cofactors, amino acids, and polyamines and several systems of human gut MAG bacteria of cluster 1 harboring a *bai* operon. Major metabolic pathways and systems are distinguished by shading colors, and unidentified but likely metabolic pathways are indicated with dotted blue arrows.

To understand the metabolic and physiological features of cluster 1 MAG bacteria harboring a complete *bai* operon, the metabolic pathways of BAs, carbohydrates, vitamins and cofactors, amino acids and polyamines, secretion systems, ATPase and electron transport systems, transport systems, and two-component systems of MAGs were investigated (Fig. 9). No bile salt hydrolase (BSH) genes were identified in any known BA 7α-dehydroxylating bacteria, including *C*. *scindens*, *C. hylemonae*, *P. sphenisci*, and *Dorea* sp., except for *C. hiranonis*, suggesting that the bile salt hydrolase of BA 7α-dehydroxylating bacteria is not common. However, all 41 MAGs harbored a complete *bai* operon involved in the conversion of primary BAs to secondary BAs as well as a gene for a BSH catalyzing the deconjugation of conjugated primary BAs, suggesting that MAG bacteria of cluster 1 are likely to use deconjugated primary BAs as well as conjugated primary BAs for BA 7α-dehydroxylation. No signal peptide sequence was identified from BSH proteins in MAG bacteria of cluster 1; therefore, they may directly take up conjugated primary BAs through unknown transporter systems into cells and then convert them to secondary BAs after deconjugation within cells (Fig. 9). With these characteristics, BA 7α-dehydroxylating bacteria of cluster 1 may be more competitive in the production of secondary BAs compared with other BA 7α-dehydroxylating bacteria, and thus, they may be mainly responsible for BA 7α-dehydroxylation in the human gut. Efficient BA 7α-dehydroxylation for the regeneration of NAD^+^ coupled to the anaerobic carbon metabolism in MAG bacteria of cluster 1 may be an important metabolic feature for their successful survival in the human gut (35).

The reconstructed metabolic pathways revealed that genes associated with the metabolism of diverse carbon sources, including glucose, galactose, fructose, mannose, maltose, xylose, citrate, and formate, were identified in all MAGs, demonstrating their diverse carbohydrate metabolic capabilities. All MAGs of cluster 1 harbored complete glycolysis and gluconeogenesis pathways, acetyl-CoA production from pyruvate by pyruvate:ferredoxin oxidoreductase, and an incomplete tricarboxylic acid (TCA) cycle lacking succinyl-CoA synthetase and malate dehydrogenase, implying that MAG bacteria of cluster 1 may have an anaerobic heterotrophic metabolic feature. The metabolic pathways also showed that MAG bacteria of cluster 1 are likely to employ an incomplete pentose phosphate pathway lacking enzymes converting glucose-6-phosphate to ribulose-5-phosphate and transaldolase to metabolize only one five-carbon sugar, xylose. Cluster 1 MAGs harbored an incomplete Wood-Ljungdahl pathway, lacking formate dehydrogenase and carbon monoxide (CO) dehydrogenase to produce acetate through acetyl-CoA with the consumption of NADH, as do reductive acetogens. Because genes associated with the production of formate and CO were not identified from the cluster 1 MAGs, MAG bacteria are likely to use formate and CO produced by other gut microbes (57). The analysis also showed that MAG bacteria are likely to produce lactate, which may be an important way to regenerate NAD^+^ with BA 7α-dehydroxylation in the cluster 1 MAG bacteria. However, MAG bacteria are not likely to produce other short-chain fatty acids, such as butyrate and propionate, that are produced by many other gut microbes (58).

The genomic analysis of MAGs showed that MAG bacteria of cluster 1 are likely to employ F- and V-type Na^+^ F_1_F_o_ ATP synthases driven by sodium motive force instead of proton motive force for ATP synthesis (Fig. 9). Because H^+^-driven ATP synthase was not identified from cluster 1 MAGs, they are likely to rely mostly on sodium motive force for ATP synthesis. In fact, cluster 1 MAGs harbored an *rnf* gene cluster (*rnfABCDEG*), potentially encoding ferredoxin:NAD^+^ oxidoreductase complex, which is likely to generate an electrochemical sodium ion gradient across the membrane that is then used to synthesize ATP (59). The incomplete Wood-Ljungdahl pathway also may be coupled to the generation of sodium motive force across the cytoplasmic membrane (60). A P-type ATPase-coding gene cluster able to export copper ion to the outside of the cell using ATP was also identified from MAGs of cluster 1.

MAGs of cluster 1 harbored a complete gene set for the biosynthesis of thiamine, which suggests that the bacteria are likely to be important thiamine producers in the human gut. Pathways for the biosynthesis of some amino acids such as threonine, cysteine, asparagine, tyrosine, and phenylalanine were identified, but the biosynthetic pathways for other amino acids, such as lysine, methionine, histidine, and valine, were not identified, meaning that availability of the latter amino acids is necessary for growth. The human gut may be an extreme environment due to high concentrations of BAs and fluctuating nutritional conditions. MAGs of cluster 1 harbored many transport systems exchanging diverse compounds and two-component systems sensing various environmental stresses and changes, which may allow MAG bacteria of cluster 1 to adapt to human gut environments for survival. An ABC transport system to export bacitracin was identified from all cluster 1 MAGs, which suggests that these bacteria may be resistant to bacitracin and that selection on bacitracin could be used as a strategy for their isolation. Genomic and metabolic analyses of cluster 1 BA 7α-dehydroxylating bacteria based on MAGs will help enable their successful cultivation, provide better understanding of their metabolic and physiological features, and contribute to precise therapeutic application for various human diseases.

### Conclusions.

In this study, uncultured BA 7α-dehydroxylating bacteria affiliated with the family *Oscillospiraceae* and mainly responsible for converting primary BAs to secondary BAs in the human gut were identified from human gut metagenomes through various bioinformatic analyses, including SSN, GNN, and relative abundance analysis of *baiE* genes, against human gut metagenome data. Surprisingly, unlike the 7α-dehydroxylating bacteria of other BaiE clusters, the abundance of cluster 1 BA 7α-dehydroxylating bacteria was significantly negatively correlated with IBD, including CD and UC, as well as advanced NAFLD, LC, and AS, and it was not significantly correlated with mild NAFLD, CRC, and ACVD, although it has been reported that secondary BAs in the human gut may be causative agents for the occurrence or development of NAFLD, LC, AS, CRC, and ACVD. Therefore, future studies should strive to obtain pure cultures of the BA 7α-dehydroxylating bacteria of cluster 1 to better understand their metabolic and physiological features and confirm their association with various human diseases, which will contribute to precise diagnostic and therapeutic application to benefit human health. Further, the approaches used in this study can be generally used for the investigation of key microbiota responsible for other important metabolisms in the human gut.

## MATERIALS AND METHODS

### Selection of query BaiE sequences.

Four BaiE protein sequences (UniProt accession numbers P19412, B0NI18, B4YSU1, and Q9RB47) were used as initial query sequences. Putative BaiE sequences were searched through BLASTP using the initial query sequences against the UniRef100 database (61), with cutoff values of 50% identity and 90% query coverage. SSNs of the resulting putative BaiE sequences were generated using the Enzyme Function Initiative Enzyme Similarity Tool (EFI-EST) based on an alignment score of 70 (approximately 90% amino acid similarity) (62). BaiE protein function of each cluster in the SSNs was inferred by GNN analysis in the contigs containing the resulting protein sequences with reference to known bile acid metabolic operons using the EFI Genome Neighborhood Tool (29, 33, 34). All sequences of clusters that were inferred to be BaiE proteins were used as query sequences for the second round of BLASTP searching of putative BaiE sequences against the UniRef databases. SSN construction from the resulting sequences and inference of BaiE protein functionality were performed as described above and all sequences of clusters inferred as putative BaiE were used as query sequences for the third-round BLASTP search. This process was repeated until no newer putative BaiE sequences were found. BaiE sequences that were experimentally verified and all putative BaiE sequences derived from the UniRef100 database were used as query sequences for searching BaiE sequences from human gut metagenomes.

### Identification of putative BaiE sequences from human gut assembly metagenomes.

Whole-genome shotgun assembly sequences of human gut microbiomes derived from the gastrointestinal tract, including the ileum, terminal ileum, colon, appendix, ascending colon, transverse colon, descending colon, sigmoid colon, rectum, and feces, available in the HMP WGS-PP1 data set were downloaded from the HMP portal (https://portal.hmpdacc.org/), and their open reading frames (ORFs) were predicted using the EMBOSS getorf software (http://emboss.sourceforge.net/). Putative BaiE sequences were identified by BLASTP using query sequences shown in Table S2 against predicted ORF sequences, with cutoff values of 30% identity and 90% query coverage. SSN construction from the identified sequences, the functional inference of each cluster as BaiE sequences, and next-round BLASTP searches against the predicted ORF sequences were performed as described above. This process was repeated until no newer putative BaiE sequences were found. The SSNs of all query and identified protein sequences were generated using EFI-EST with an alignment score of 70 and visualized using Cytoscape v.3.3 (63), as described previously (64).

### Phylogenetic and GNN analyses of identified protein sequences.

The query and resulting protein sequences were aligned using the web-based tool Clustal Omega (https://www.ebi.ac.uk/Tools/msa/clustalo/), and a phylogenetic tree with bootstrap values (1,000 replicates) based on the maximum-likelihood algorithm was constructed using MEGA7 software (65). Genomes or contigs containing genes encoding protein sequences of each cluster in the SSNs were investigated, and GNN analysis in genomes or contigs was performed to infer their function as BaiE proteins capable of converting primary to secondary BAs. Where genomes (or contigs containing genes encoding protein sequences) were not identified, GNN analyses were performed in the genomes derived from uncultured human gut bacterial genome databases that contained their highest sequence identity (66–69). All possible ORFs of operons containing protein-coding sequences were predicted by the Prodigal program (70), and their gene functions were annotated by eggNOG-mapper version 4.5.1 (71).

### Overexpression and activity assay of putative *baiA* and *baiB* genes in cluster 1.

For overexpressing the putative *baiA* (DER43_01330) and *baiB* (DER43_01335) genes in the uncultured *Clostridiales* bacterium UBA11811, two nucleotides, 5′-GGAATTCCAT-*baiA*-AAGCTTGGG-3′ and 5′-GGAATTCCAT-*baiB*-GAATTCC-3′, were synthesized by Macrogen (South Korea); doubled-digested with NdeI/HindIII and NdeI/EcoRI, respectively; and then cloned into the pET-28a vector (Novagen, USA). Escherichia coli BL21(DE3) cells harboring plasmids pET28a, pET28a-*baiA*, and pET28a-*baiB* were aerobically cultured to an optical density of approximately 0.6 at 600 nm in 100 mL LB broth containing kanamycin (50 μg/mL) at 37°C. Next, 1 mM isopropyl-β-d-thiogalactopyranoside (IPTG) was added to the cultures, and the E. coli cells were incubated overnight at 16°C. E. coli cells were harvested by centrifugation and resuspended in 10 mL lysis buffer (50 mM HEPES, 300 mM KCl, 4 mM imidazole, 10 mM β-mercaptoethanol, and 10% glycerol; pH 7.5). Crude cell extracts that were prepared by disrupting the cells by sonication and removing cell debris by centrifugation were loaded onto a nickel-nitrilotriacetic acid (Ni-NTA) affinity column equilibrated with lysis buffer. The protein bounded columns were eluted sequentially with 5 column volumes of elution buffer (50 mM HEPES, 300 mM KCl, and 10% glycerol; pH 7.5) containing 5 mM, 10 mM, and 300 mM imidazole. Protein fractions eluted by elution buffer containing 300 mM imidazole were desalted and concentrated using a 10,000-molecular-weight-cutoff (MWCO) Pierce PES protein concentrator (Thermo Scientific, USA), following the manufacturer’s protocol.

Enzyme assays were conducted in 1.0-mL reaction mixtures containing 50 mM HEPES (pH 7.5), 50 mM KCl, 200 μM NAD^+^, 100 μM coenzyme A sodium salt hydrate, 200 μM ATP, and 20 μM sodium cholate at 37°C for 24 h under anaerobic conditions. The enzyme reactions were initiated by individually adding purified putative BaiB protein, purified putative BaiB and BaiA protein mixture, and E. coli cell extract with pET28a (negative control) to the reaction mixtures. The reactions were stopped by the addition of an equal volume of 100% acetone, and two reaction products, cholyl-CoA and 3-oxo-cholyl-CoA, were analyzed using a liquid chromatography quadrupole time-of-flight mass spectrometry (LC-Q-TOF-MS) system (Agilent Technologies, USA) equipped with a 1290 Infinity ultra-high-performance liquid chromatograph, 6550 iFunnel Q-TOF-MS, and ZORBAX SB-C_18_ column (2.1 mm by 50 mm, 1.8 μm), as described previously (72). Water containing 10 mM ammonium acetate (pH 9.0) (A) and acetonitrile (B) was used as the mobile phases at a flow rate of 0.3 mL/min with the following gradients: 0 to 17 min, 5 to 95% B; 17 to 18 min, 95 to 5% B; and 18 to 20 min, 5% B. Mass spectrometry was performed under the following conditions: polarity, positive; gas temperature, 250°C; nebulizer, 35 lb/in^2^; capillary, (+) 4,000 V; MS range, 50 to 1,600 *m/z*; and MS/MS range, 50 to 1,600 *m/z*. Cholyl-CoA and 3-oxo-cholyl-CoA in the total LC-Q-TOF-MS data were searched using the “find by formular” function (Agilent MassHunter Qualitative Analysis B10.0).

### Abundance analysis of *baiE* genes and *baiE* gene transcripts of BaiE clusters in human gut microbiota.

The relative abundance of *baiE* genes and *baiE* gene transcripts of clusters inferred as BaiE clusters through the GNN analysis in human gut metagenomes was estimated as follows. In brief, human gut metagenome and metatranscriptome data (Table S4 in the supplemental material) were downloaded from GenBank Sequence Read Archive, and their sequencing reads were trimmed at a quality threshold of 30 and minimum length of 50 bp using Sickle (https://github.com/najoshi/sickle). High-quality sequencing reads were matched to all nucleotide sequences of each BaiE cluster using Burrows-Wheeler Aligner (BWA) (http://bio-bwa.sourceforge.net), based on the criteria of best match with a 90% minimum identity and 20-bp minimum alignment, and the sequencing reads matched to each BaiE cluster were counted using BEDtools (https://bedtools.readthedocs.io/). The read counts of *baiE* genes and *baiE* gene transcripts in gut metagenome and metatranscriptome samples were normalized based on read counts per million sequencing reads and visualized by box plots using the ggplot2 package (v3.1.0) in R program. The *baiE* gene and transcript abundances of each BaiE cluster in the human gut microbiota were determined using all data listed in Table S4, while the *baiE* gene abundance of each BaiE cluster according to disease cases (CD, UC, CRC, ACVD, NAFLD, LC, AS, and T2DM) in the human gut microbiota was determined using only human gut metagenome cohort data.

10.1128/msystems.00455-22.6 TABLE S4 Human gut metagenome and metatranscriptome data used for the estimation of *baiE* gene and transcript abundances of each BaiE cluster. Download Table S4, PDF file, 0.1 MB. Copyright © 2022 Kim et al. 2022 Kim et al. https://creativecommons.org/licenses/by/4.0/ This content is distributed under the terms of the Creative Commons Attribution 4.0 International license.

### Correlation analysis between *baiE* gene abundance and bile acid level.

The human gut metagenome (NCBI SRA database BioProject number PRJNA400072 and DDBJ Sequence Read Archives numbers DRA006684 and DRA008156) and metabolome (National Institutes of Health Common Fund’s Metabolomics Data Repository PR000677; also, see the supplemental material) data sets generated by Franzosa et al. (73) and Yachida et al. (74) were download. By using only healthy-control metagenome and metabolome data sets, Spearman correlations between *baiE* gene abundances (all sequences and *baiE* clusters 1, 4, and 6) and the ratios of secondary BAs (DCA and LCA) and primary BAs (CA and CDCA) were calculated, and the results were visualized using the R ggpubr package (https://rpkgs.datanovia.com/ggpubr/index.html).

### Phylogenetic and genomic analysis of cluster 1 bacteria harboring *baiE* genes.

To more comprehensively understand the phylogenetic and genomic features of BA 7α-dehydroxylating bacteria belonging to cluster 1, a BLASTP search was performed using the BaiE protein sequences of cluster 1 against human gut MAG databases (66–69). From MAGs harboring cluster 1 *baiE* genes identified from databases and genomes of type strains classified as members of the class *Clostridia* by the Genome Taxonomy DataBase (GTDB; http://gtdb.ecogenomic.org/), 120 common marker genes were extracted and their protein sequences were concatenated and aligned using the GTDB-tk program (https://github.com/Ecogenomics/GTDBTk). A phylogenetic tree of the aligned concatenated protein sequences was constructed using the neighbor-joining algorithm in the MEGA7 program and pruned using the Interactive Tree Of Life (75). ANI values between MAGs were calculated using a stand-alone program, the Orthologous Average Nucleotide Identity Tool (http://www.ezbiocloud.net/sw/oat) (76). Gene annotations of MAGs harboring *baiE* genes of cluster 1 were performed using the eggNOG-mapper software, the KEGG automatic annotation server (https://www.genome.jp/kegg/kaas/) (77), and Prokka (78), and their metabolic pathways were reconstructed based on predicted pathways and gene annotations. In addition, the presence or absence of the pathways and transport and sensing systems in each MAG was confirmed through BLASTP analyses against each MAG, using reference protein sequences available in other closely related bacteria.

### Data availability.

The data that support the findings of this study are available in National Center for Biotechnology Information Sequence Read Archive (https://www.ncbi.nlm.nih.gov/sra) with BioProject ID numbers PRJNA275349, PRJNA48479, PRJNA354235, PRJEB2054, PRJEB5224, PRJNA389280, PRJNA447983, PRJEB27928, PRJEB12449, PRJEB7774, PRJEB6070, PRJNA389927, PRJEB10878, PRJNA453965, PRJEB21528, PRJNA373901, PRJEB6337, PRJNA375935, PRJNA422434, and PRJEB7759.

10.1128/msystems.00455-22.7 TABLE S5 Mouse gut metagenome data used for the estimation of *baiE* gene abundance of each BaiE clusters in the mouse gut microbiota. Download Table S5, PDF file, 0.1 MB. Copyright © 2022 Kim et al. 2022 Kim et al. https://creativecommons.org/licenses/by/4.0/ This content is distributed under the terms of the Creative Commons Attribution 4.0 International license.
