# Supplementary material for: Identification and Characterization of Major Bile Acid 7α-Dehydroxylating Bacteria in the Human Gut
Source: mSystems. 2022 Jun 23;7(4):e00455-22. doi: 10.1128/msystems.00455-22 (PMC9426597; doi:10.1128/msystems.00455-22)
Supplement: TABLE S1 [file msystems.00455-22-s0003.pdf]

TABLE S1

UniProt acc. no.	Organism	Reference
P19412	*Clostridium scindens* VPI 12708	1
B0NI18	*Clostridium scindens* ATCC 35704	2
B4YSU1	*Clostridium hylemonae* DSM 15053	3
Q9RB47	*Clostridium hiranonis* DSM 13275	4

References

1. Dawson JA, Mallonee DH, Björkhem I, Hylemon PB. 1996. Expression and characterization of a C_{24} bile acid 7α-dehydratase from *Eubacterium* sp. strain VPI 12708 in *Escherichia coli*. *J Lipid Res* 37:1258–1267.
2. Bhowmik S, Chiu HP, Jones DH, Chiu HJ, Miller MD, Xu Q, Farr CL, Ridlon JM, Wells JE, Elsliger MA. 2016. Structure and functional characterization of a bile acid 7α dehydratase BaiE in secondary bile acid synthesis. *Proteins* 84:316–331.
3. Ridlon JM, Kang DJ, Hylemon PB. 2010. Isolation and characterization of a bile acid inducible 7α-dehydroxylating operon in *Clostridium hylemonae* TN271. *Anaerobe* 16:137–146.
4. Wells JE, Hylemon PB. 2000. Identification and characterization of a bile acid 7α-dehydroxylation operon in *Clostridium* sp. strain TO-931, a highly active 7α-dehydroxylating strain isolated from human feces. *Appl Environ Microbiol* 66: 1107–1113.
