# Supplementary material for: Identification and Characterization of Major Bile Acid 7α-Dehydroxylating Bacteria in the Human Gut
Source: mSystems. 2022 Jun 23;7(4):e00455-22. doi: 10.1128/msystems.00455-22 (PMC9426597; doi:10.1128/msystems.00455-22)
Supplement: TABLE S2 [file msystems.00455-22-s0004.pdf]

TABLE S2

UniProt acc. no.	Functional description	Taxonomic description*
F7KMI9	Bile acid 7α dehydratase	[U]*Lachnospiraceae* bacterium 5_1_57FAA
A0A1C5W919		[U]*Ruminococcus* sp.
A0A0K9NDZ1		*Dorea* sp. D27
A0A3D1VEZ9	Nuclear transport factor 2 family protein	[U]*Clostridiales* bacterium UBA11811
A0A3R5Y295		*Clostridium* sp. AF15-17LB
A0A395YCM0		*Dorea* sp. AM58-8
UPI000E527D25		*Peptoclostridium* sp. AF21-18
UPI000471FD15		[U]*Clostridiales* bacterium VE202-26
UPI0008379CD3		*Clostridium* sp. Marseille-P2538
UPI00048D1F9B		*Proteocatella sphenisci* DSM 23131
R5AU51	Uncharacterized protein	[U]*Firmicutes bacterium* CAG:103

* [U], uncultured
