# Supplementary material for: Identification and Characterization of Major Bile Acid 7α-Dehydroxylating Bacteria in the Human Gut
Source: mSystems. 2022 Jun 23;7(4):e00455-22. doi: 10.1128/msystems.00455-22 (PMC9426597; doi:10.1128/msystems.00455-22)
Supplement: TABLE S3 [file msystems.00455-22-s0005.pdf]

TABLE S3

SRS075078, SRS063985, SRS056519, SRS015578, SRS142890, SRS049896, SRS045528, SRS019161, SRS018427, SRS015133, SRS017521, SRS045713, SRS147652, SRS022071, SRS050752, SRS017307, SRS101433, SRS013951, SRS015782, SRS020328, SRS050925, SRS012273, SRS098717, SRS018351, SRS024435, SRS048870, SRS065504, SRS015264, SRS014979, SRS043411, SRS021484, SRS022524, SRS078176, SRS042628, SRS019030, SRS064276, SRS049995, SRS014613, SRS015065, SRS019601, SRS014235, SRS015960, SRS011302, SRS014923, SRS078419, SRS140492, SRS013098, SRS064276, SRS015578, SRS142890, SRS014923, SRS101433, SRS147652, SRS042628, SRS045713, SRS014613, SRS078419, SRS021484, SRS017307, SRS065504, SRS012273, SRS016954, SRS056519, SRS063985, SRS022524, SRS075078, SRS047014, SRS015960, SRS140492, SRS011302, SRS014979, SRS019601, SRS015133, SRS098717, SRS014235, SRS022071, SRS045528, SRS050752, SRS050925, SRS019685, SRS013951, SRS019161, SRS014235, SRS142503, SRS024435, SRS103987, SRS013687, SRS143598, SRS015663, SRS011529
