# Supplementary figures and images for: Identification and Characterization of Major Bile Acid 7α-Dehydroxylating Bacteria in the Human Gut
Source: mSystems. 2022 Jun 23;7(4):e00455-22. doi: 10.1128/msystems.00455-22 (PMC9426597; doi:10.1128/msystems.00455-22)

FIG S1

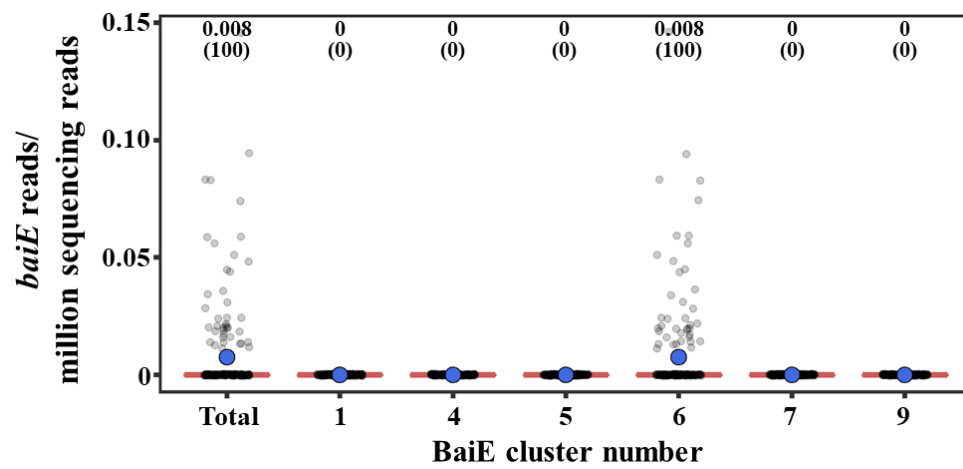

Supplement: FIG S1 [file msystems.00455-22-s0001.pdf]

**FIG S2**

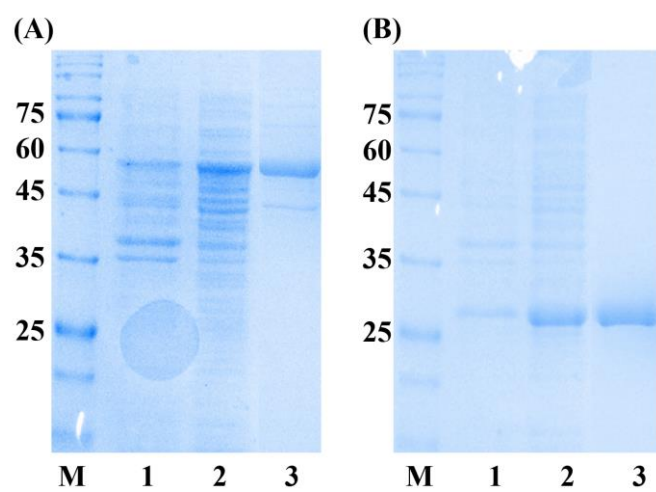

Supplement: FIG S2 [file msystems.00455-22-s0002.pdf]
