# Supplementary material for: Identification and Characterization of Major Bile Acid 7α-Dehydroxylating Bacteria in the Human Gut
Source: mSystems. 2022 Jun 23;7(4):e00455-22. doi: 10.1128/msystems.00455-22 (PMC9426597; doi:10.1128/msystems.00455-22)
Supplement: TABLE S4 [file msystems.00455-22-s0006.pdf]

TABLE S4

GenBank Bioproject no.	Disease case* (no. of samples)	Country	Reference
PRJNA275349	Healthy (253)	USA	1
PRJNA48479	Healthy (253)	USA	1
PRJNA354235[†, ‡]	Healthy (308)	USA	2
PRJEB2054	Healthy (85)/CD & UC (39)	Denmark & Spain	3
PRJEB5224	Healthy (109)/CD & UC (103)	Denmark & Spain	4
PRJNA389280	Healthy (65)/CD & UC (235)	USA	5
PRJNA447983	Healthy (28)/CRC (32)	Italy	6
PRJEB27928	Healthy (60)/CRC (22)	Germany	6
PRJEB12449	Healthy (52)/CRC (52)	USA	7
PRJEB7774	Healthy (63)/AD & CRC (93)	Austria	8
PRJEB6070	Healthy (66)/AD & CRC (133)	France & Germany	9
PRJNA389927	Healthy (28)/AD & CRC (56)	USA & Canada	10
PRJEB10878	Healthy (53)/CRC (75)	China	11
PRJNA453965	Healthy (71)/BC (62)	China	12
PRJEB21528	Healthy (171)/ACVD (214)	China	13
PRJNA373901[†]	Mild NAFLD (74) & advanced NAFLD (12)	USA	14
PRJEB6337	Healthy (114)/LC (123)	China	15
PRJNA375935	Healthy (114)/AS (97)	China	16
PRJNA422434	Healthy (183)/T2DM (187)	China	17

*Abbreviations: CD, Crohn's disease; UC, ulcerative colitis; CRC, colorectal cancer; AD, adenoma; BC, breast cancer; ACVD, atherosclerotic cardiovascular disease; NAFLD, non-alcoholic fatty liver disease; LC, liver cirrhosis.

[†]The *baiE* gene abundance in NAFLD was compared with those in the healthy samples of PRJNA354235.

[‡]Only bioproject PRJNA354235 includes metatranscriptome data of human gut microbiota, which were used for the estimation of *baiE* gene transcripts of each clusters.

References

1. Lloyd-Price J, Arze C, Ananthakrishnan AN, Schirmer M, Avila-Pacheco J, Poon TW, Andrews E, Ajami NJ, Bonham KS, Brislawn CJ. 2019. Multi-omics of the gut microbial ecosystem in inflammatory bowel diseases. *Nature* 569:655–662.
2. Abu-Ali GS, Mehta RS, Lloyd-Price J, Mallick H, Branck T, Ivey KL, Drew DA, DuLong C, Rimm E, Izard J. 2018. Metatranscriptome of human faecal microbial communities in a cohort of adult men. *Nat Microbiol* 3:356–366.
3. Qin J, Li R, Raes J, Arumugam M, Burgdorf KS, Manichanh C, Nielsen T, Pons N, Levenez F, Yamada T. 2010. A human gut microbial gene catalogue established by metagenomic sequencing. *Nature* 464:59–65.
4. Li J, Jia H, Cai X, Zhong H, Feng Q, Sunagawa S, Arumugam M, Kultima JR, Prifti E, Nielsen T. 2014. An integrated catalog of reference genes in the human gut microbiome. *Nat Biotechnol* 32:834–841.
5. Schirmer M, Franzosa EA, Lloyd-Price J, McIver LJ, Schwager R, Poon TW, Ananthakrishnan AN, Andrews E, Barron G, Lake K. 2018. Dynamics of metatranscription in the inflammatory bowel disease gut microbiome. *Nat Microbiol* 3:337–346.
6. Thomas AM, Manghi P, Asnicar F, Pasolli E, Armanini F, Zolfo M, Beghini F, Manara S, Karcher N, Pozzi C. 2019. Metagenomic analysis of colorectal cancer datasets identifies cross-cohort microbial diagnostic signatures and a link with choline degradation. *Nat Med* 25:667–678.
7. Vogtmann E, Hua X, Zeller G, Sunagawa S, Voigt AY, Hercog R, Goedert JJ, Shi J, Bork P, Sinha R. 2016. Colorectal cancer and the human gut microbiome: reproducibility with whole-genome shotgun sequencing. *PLoS One* 11:e0155362.
8. Feng Q, Liang S, Jia H, Stadlmayr A, Tang L, Lan Z, Zhang D, Xia H, Xu X, Jie Z. 2015. Gut microbiome development along the colorectal adenoma–carcinoma sequence. *Nat Commun* 6:6528.
9. Zeller G, Tap J, Voigt AY, Sunagawa S, Kultima JR, Costea PI, Amiot A, Böhm J, Brunetti F, Habermann N. 2014. Potential of fecal microbiota for early-stage detection of colorectal cancer. *Mol Syst Biol* 10:766.
10. Hannigan GD, Duhaime MB, Ruffin MT 4th, Koumpouras CC, Schloss PD. 2018. Diagnostic potential and interactive dynamics of the colorectal cancer virome. *MBio* 9:e02248–18.

11. Yu J, Feng Q, Wong SH, Zhang D, Liang QY, Qin Y, Tang L, Zhao H, Stenvang J, Li Y. 2017. Metagenomic analysis of faecal microbiome as a tool towards targeted non-invasive biomarkers for colorectal cancer. *Gut* 66:70–78.
12. Zhu J, Liao M, Yao Z, Liang W, Li Q, Liu J, Yang H, Ji Y, Wei W, Tan A. 2018. Breast cancer in postmenopausal women is associated with an altered gut metagenome. *Microbiome* 6:136.
13. Jie Z, Xia H, Zhong SL, Feng Q, Li S, Liang S, Zhong H, Liu Z, Gao Y, Zhao H. 2017. The gut microbiome in atherosclerotic cardiovascular disease. *Nat Commun* 8:845.
14. Loomba R, Seguritan V, Li W, Long T, Klitgord N, Bhatt A, Dulai PS, Caussy C, Bettencourt R, Highlander SK. 2017. Gut microbiome-based metagenomic signature for non-invasive detection of advanced fibrosis in human nonalcoholic fatty liver disease. *Cell Metab* 25:1054–1062.
15. Qin N, Yang F, Li A, Prifti E, Chen Y, Shao L, Guo J, Le Chatelier E, Yao J, Wu L. 2014. Alterations of the human gut microbiome in liver cirrhosis. *Nature* 513:59–64.
16. Wen C, Zheng Z, Shao T, Liu L, Xie Z, Le Chatelier E, He Z, Zhong W, Fan Y, Zhang L. 2017. Quantitative metagenomics reveals unique gut microbiome biomarkers in ankylosing spondylitis. *Genome Biol* 18:142.
17. Qin J, Li Y, Cai Z, Li S, Zhu J, Zhang F, Liang S, Zhang W, Guan Y, Shen D. 2012. A metagenome-wide association study of gut microbiota in type 2 diabetes. *Nature* 490:55–60.
