# Supplementary material for: Identification and Characterization of Major Bile Acid 7α-Dehydroxylating Bacteria in the Human Gut
Source: mSystems. 2022 Jun 23;7(4):e00455-22. doi: 10.1128/msystems.00455-22 (PMC9426597; doi:10.1128/msystems.00455-22)
Supplement: TABLE S5 [file msystems.00455-22-s0007.pdf]

TABLE S5

GenBank Bioproject no.	Mouse strain	No. of samples	Reference
PRJEB7759	129S	8	1
	BALB/c	8	
	C57/BL6	100	
	NOD	8	
	SJL	8	
	SJL-C57BL/6	8	
	SV129	34	
	Swiss web	10	

Reference

1. Xiao L, Feng Q, Liang S, Sonne SB, Xia Z, Qiu X, Li X, Long H, Zhang J, Zhang D. 2015. A catalog of the mouse gut metagenome. *Nat Biotechnol* 33:1103–1108.
